# In vivo studies of genomic packaging in the dsRNA bacteriophage Φ8

**DOI:** 10.1186/1471-2180-5-10

**Published:** 2005-03-11

**Authors:** Jian Qiao, Xueying Qiao, Leonard Mindich

**Affiliations:** 1 Department of Microbiology, The Public Health Research Institute. Newark, New Jersey 07103, USA

## Abstract

**Background:**

Φ8 is a bacteriophage containing a genome of three segments of double-stranded RNA inside a polyhedral capsid enveloped in a lipid-containing membrane. Plus strand RNA binds and is packaged by empty procapsids. Whereas Φ6, another member of the Cystoviridae, shows high stringency, serial dependence and precision in its genomic packaging *in vitro *and *in vivo*, Φ8 packaging is more flexible. Unique sequences (*pac*) near the 5' ends of plus strands are necessary and sufficient for Φ6 genomic packaging and the RNA binding sites are located on P1, the major structural protein of the procapsid.

**Results:**

In this paper the boundaries of the Φ8 *pac *sequences have been explored by testing the *in vivo *packaging efficacy of transcripts containing deletions or changes in the RNA sequences. The *pac *sequences have been localized to the 5' untranslated regions of the viral transcripts. Major changes in the *pac *sequences are either tolerated or ameliorated by suppressor mutations in the RNA sequence. Changes in the genomic packaging program can be established as a result of mutations in P1, the major structural protein of the procapsid and the determinant of RNA binding specificity.

**Conclusion:**

Although Φ8 is distantly related to bacteriophage Φ6, and does not show sequence similarity, it has a similar genomic packaging program. This program, however, is less stringent than that of Φ6.

## Background

The Cystoviridae are a family of bacteriophages having genomes consisting of three segments of double-stranded RNA (dsRNA). The RNA is contained within a polyhedral capsid which is enveloped in a lipid-containing membrane [1]. Φ6 was the first and the most thoroughly studied member of this family. The packaging of the Φ6 genome was found to progress through a program that involves the sequential packaging of the plus strands of segments S, M and L in that order [2]. This program is rather stringent for Φ6 in that segment S can be packaged alone while M requires prior packaging of S and L requires prior packaging of M. Segment L can be packaged to some extent with only prior packaging of S but this is much less efficient than the normal packaging [3,4]. The Cystoviridae are the only family of RNA viruses that are reliably known to package their genomic segments into preformed capsids. Although the Reoviridae have an inner core particle structure strikingly similar to that of the Cystoviridae, their mechanisms of genomic packaging have not yet been determined [5]. Packaging in the Cystoviridae is driven by an NTPase motor comprised of a hexamer of protein P4 [6-8]. The plus strands of Φ6 have an 18 base consensus sequence at the 5' end and at about 50 bases downstream, a sequence of about 200 nucleotides that is necessary and sufficient for packaging into procapsids. The 200 nucleotide sequence is called *pac *and it is unique for each segment, with very limited identity in sequence between the three segments. Minus strand synthesis begins after the completion of the plus strand packaging and plus strand synthesis begins after minus strand synthesis is completed. A model has been proposed that involves the programmed changes in the binding sites for plus strands on the surface of the procapsid as a function of the amount of RNA in the particle [2].

The *pac *sequences of Φ6 were defined by deletion analysis of the plus strand transcripts of plasmids carrying cDNA copies of the genomic segments. In vitro packaging was used to assess the success of packaging of transcripts harboring a series of deletions. It was found that the *pac *sequences ended about 50 nucleotides before the first open reading frames (orfs) of the segments [9]. The *pac *sequences showed considerable secondary structure, with a number of stem-loops [10]. Comparison of the *pac *sequences of close relatives of Φ6 suggested that the stem-loop structures were important because base changes preserved the stems in a number of cases [2]. Φ8 is the most distantly related member of the Cystoviridae relative to Φ6. It has no sequence identity or similarity to Φ6 and it has significant differences in genetic and physical structure [11,12]. Our aim was to determine whether Φ8 genomic packaging uses *pac *sequences in a manner similar to that found for Φ6. Whereas the *pac *sequences were defined in Φ6 by in vitro packaging studies, we have found that in vitro packaging for Φ8 is non-specific when evaluated by the uptake of radioactive RNA [12]. We have therefore used in vivo transcript acquisition to define the *pac *sequences of Φ8 in this report.

## Results

### The determination of the *pac *sequences of Φ8

**Figure 1:**
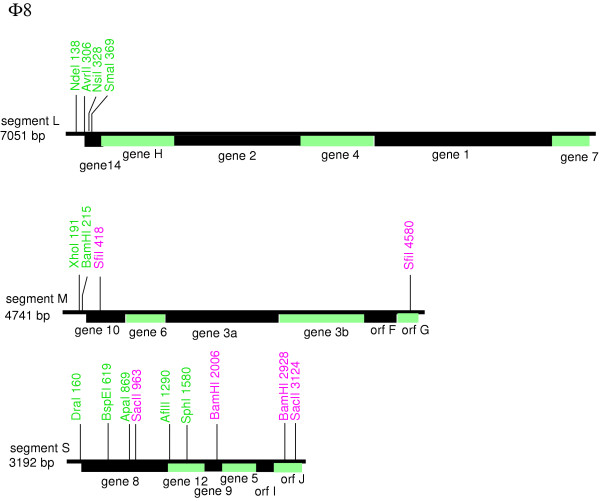
cDNA map of the genomic segments of bacteriophage Φ8. An SP6 promoter precedes the cDNA copies in the plasmids.

The *pac *sequences of Φ6 were determined by deletion analysis [9]. Plus strand transcripts of cDNA plasmids were prepared with deletions near the 5' ends of particular genomic segments. These RNA molecules were incubated with procapsids and the two other wild type transcripts. Minus strand synthesis was then used as the measure of packaging efficiency, since minus strand synthesis was found to be dependent upon packaging of the three genomic segments. It was found that the *pac *sequences ended about 50 nucleotides before the first open reading frames (orfs). These orf positions were at nucleotides 305, 367 and 270 for segments S, M and L respectively. In the case of Φ8 the first reading frames in segments S, M and L begin at nucleotides 188, 263 and 253 respectively [11](Fig. 1). This would put the ends of the *pac *sequences at about 140, 210 and 200 for S, M and L if the same relationship to *orf*s is operating in Φ8 as in Φ6. We have done deletion analysis on segments S, M and L and measured the efficacy of plasmid transcript acquisition into infectious phage as a measure of packaging success. Plasmid transcript acquisition is effected by the introduction of non replicating cDNA plasmids into cells by electroporation. The transcripts of the plasmids produce live phage if they are packaged and if they contain all of the necessary genes. If a particular transcript lacks a functional *pac *sequence, it will not be packaged and live phage will not be produced. With normal wild type transcripts one finds thousands of infectious centers produced. We found that the *pac *sequences are located in the 5' untranslated region of the plus strands but that they are closer to the first orfs than found for Φ6. Whereas many of the small changes in the *pac *sequences of Φ6 resulted in diminutions of the order of one hundred fold in acquisition frequency [13], changes of this type in Φ8 generally led to changes of less than ten fold (Table 3).

### Segment L

A deletion of nucleotides 244 to 328 in L does not interfere with packaging (Table 1, plasmid 3316). Although this deletion involves gene 14, the gene product is not required for plaque formation. Deletions starting at nucleotide 219 or earlier compromise packaging severely or completely (Table 1). Deletions from 329 to 745 are also tolerated if gene Hb is complemented in *trans *(not shown). The downstream end of the *pac *sequence is consequently located in the region between nucleotides 219 and 244 and therefore between 9 and 34 bases upstream of the gene 14 orf.

### Segment M

A deletion of 196 to 215 in M compromises segment acquisition; and deletion of the *BamHI *site at 215 compromises acquisition (Table 2, plasmid 3324). Creation of a HindIII site at position 238 does not reduce acquisition (Table 2, plasmid 3348), but deletion of this site does (Table 2, plasmid 3356). A deletion of bases 34 to 39 also compromises viability in M (Table 2, plasmid 3043). Therefore the downstream end of the *pac *sequence of M is located in the region of nucleotide 242 which places it either inside of gene 10 or close to it.

### Segment S

**Figure 2:**
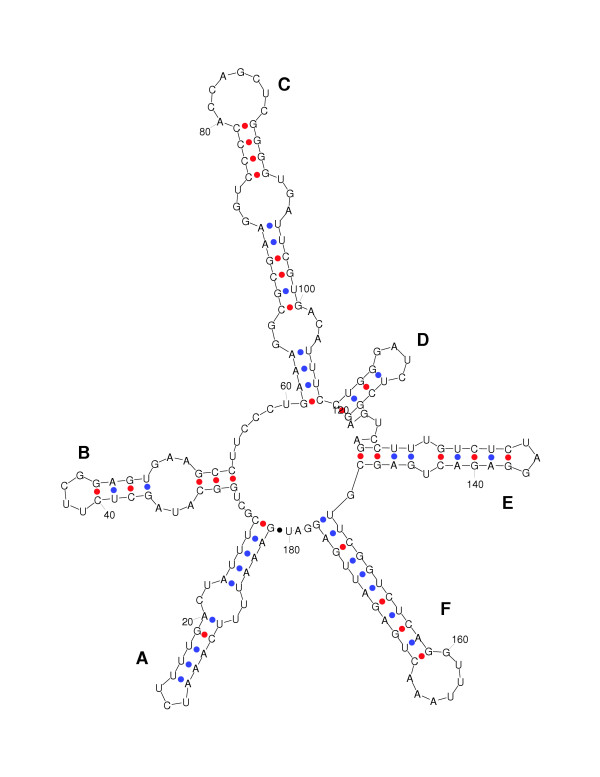
mfold 15 of the 5' region of wild type segment S plus strand. The maximum distance between paired bases is set at 50.

**Figure 3:**
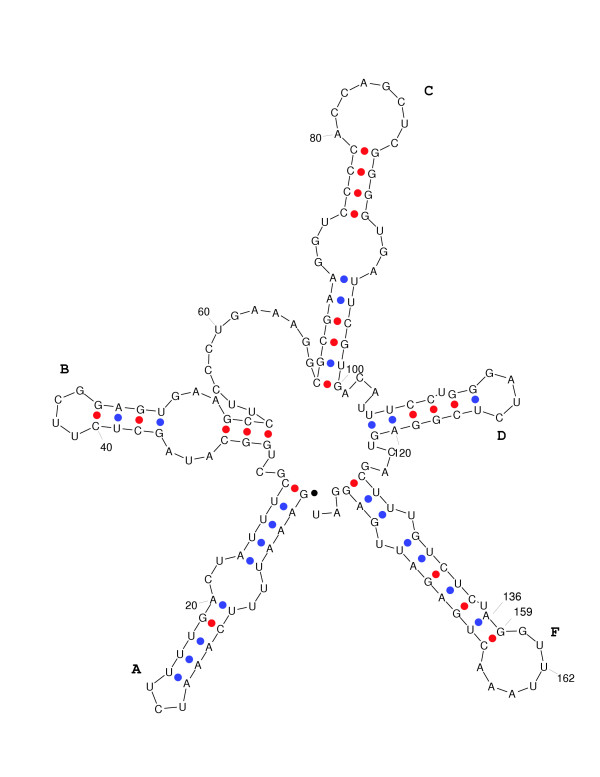
mfold 15 of the 5' region of the segment S plus strand with a deletion of bases 137 to 158 (Table 3, plasmid 2816). The bases are numbered as in the undeleted wild type segment S of Fig.2

In the case of segment S we find that a deletion of nucleotides from 137 to 158 has little effect on the formation of viable phage (Table 3, plasmid 2816). The deletion of 137 to 158 is interesting in that the stem at F (Fig. 2) is recreated and the loop, nucleotides 160 to 166, is maintained in the deletion structure (Fig. 3). Base changes and deletions in the loops of B, C, D and E do not show great diminution of packaging. Larger deletions such as 75 to 158 (Table 3, plasmid 2820) or 120 to 158 (Table 3, plasmid 2817) completely abolished plaque formation. This would place the *pac *region somewhere between nucleotides 40 and 139, which would make the *pac *region in Φ8 S about half the size of the corresponding region in Φ6. But an examination of the consequences of base changes in the exterior of this region suggests a somewhat more complicated picture. The region between nucleotides 120 and 139 is very important; but changes in the region from 160 to 170 also are significant, suggesting that the *pac *region extends from about 40 to 170. Although loop F at position 160 to 166 is maintained in the deletion of 137 to 158, the sequence of the loop can be changed (Table 3, plasmid 2939) without compromising packaging. However changing bases 159 and 160 (Table 3, plasmid 2955) prevents packaging. This suggests that the stem or the stem-loop junction may be more important than the loop. There is a stem loop structure from 125 to 147 with the loop UAG at 135 (Fig. 2). Changing the UAG to CUU has little effect (Table 3, plasmid 2914). There are two U's that are unpaired in the stem and changing them so that they pair results in no change in packaging. But other changes in the stem result in poor packaging. It appears that the stems are more sensitive than the loops in the *pac *region in terms of interference with packaging. The downstream end of the *pac *sequence is again very close to, or even within, the first orf, which in this case is gene 8 (Fig. 1).

### The consequences of replacing the *pac *sequence of M with that of L

Base changes within the *pac *regions of the Φ6 genome have resulted in the isolation of suppressor mutations in protein P1, the major structural protein of the procapsid and the determinant of the RNA binding sites. However, in the case of Φ8 we have found less stringency in the *pac *sequences and we have not found suppressor mutations in P1. In the case of Φ6 it was found that suppressor mutations in P1 could also be isolated when conditions were established that were contrary to the packaging program [14]. Packaging of segments M and L without segment S could be established in carrier states if suppressor mutations were promoted in gene 1 [14]. We had previously found that gene 8 was indispensable in Φ8, thereby making it difficult to establish carrier states in the absence of segment S. We therefore set out to select for a condition where the M segment would have the *pac *sequence of L and that phage would be selected for with both the M and L segments having the same *pac *sequences.

We therefore set out to construct a plasmid with a cDNA copy of segment M but with the pac sequence of segment L. A plasmid was prepared with the cDNA copy of segment M. It was cut with *BglII *and *XhoI *which removed the first 191 nucleotides and replaced with *BglII/SmaI *fragment of a plasmid with a cDNA copy of L. This places the first 371 nucleotides of L at the 5' end of segment M. This plasmid is called pLM3018. Electroporation of this plasmid along with plasmids containing normal copies of L and S into cells carrying plasmids expressing SP6 RNA polymerase resulted in only one plaque as opposed to tens of thousands with normal wild type constructs (Table 4). The RNA of the resulting viable phage was analyzed and it was found that the *pac *sequence of L remained on segment M. Gene 1 on segment L was cloned as cDNA and inserted into a copy of normal segment L. The electroporation experiment was repeated with the original plasmids or with the normal S, the M segment with the L *pac *sequence and the new L plasmid containing the replacement of wild type gene 1 with that of the presumed mutant gene 1. The original combination gave no plaques, while the combination with the new gene 1 gave thousands of plaques as did the new L plasmid with normal M and S (Table 4). The yield from the latter test was ten times that of the electroporation with the chimeric M. Sequencing of gene 1 showed that a single change at U4807C had occurred. Reisolation of the small fragment between sites at 4030 and 4850 confirmed that this mutation was responsible for the ability to package the chimeric M. The amino acid change generated in protein P1 by the mutation is V242A. In Φ6, all suppressor mutations involved in packaging have been in protein P1, which is the major structural protein of the procapsid and the source of the specific binding sites for the plus strands of the genomic segments.

## Discussion

Plus strands of the three genomic segments of bacteriophage Φ6 are packaged by core structures composed of proteins P1, P2, P4 and P7 [2]. They bind to protein P1, the major structural protein of the core and are translocated to the interior by hexamers of the NTPase P4. We had worked out the identification of sequences necessary and sufficient for packaging in bacteriophage Φ6 by *in vitro *packaging experiments. A region near the 5' end designated as *pac *is necessary and sufficient for packaging. We have established that the *pac *sequences of Φ6 are located in the 5' untranslated regions of the plus strands. Whereas the *in vitro *packaging of Φ6 is very stringent and specific, that of Φ8 was found to be non-specific when uptake of radioactive RNA was being assayed. *In vivo *packaging was more specific [12]. We therefore established a system of *in vivo *transcript acquisition to test the sequence limits for the packaging of the Φ8 genome.

In this report, we have identified the *pac *regions for bacteriophage Φ8, a distant relative of Φ6. All three plus strands of Φ8 have a consensus sequence of 9 nucleotides at the 5' end. Again, we have found that the *pac *regions occupy space in the 5' untranslated regions of the plus strands. Although the *pac *regions of both Φ6 and Φ8 have many stem-loop structures, there is no sequence or structural similarity between the two sets. There is also no sequence similarity between the *pac *sequences of the three genomic segments of Φ8. While the Φ6 *pac *sequences extend approximately to nucleotides 230, 305 and 270 for L, M and S respectively; those of Φ8 extend to near nucleotides 244, 242 and 170. The Φ8 *pac *sequences end closer to the first *orfs *than those of Φ6. In the cases of segments S and M the *pac *sequences might overlap the first orfs. Changes within the *pac *region of Φ8 segment S are less destructive of packaging than similar changes in *pac *regions of Φ6. Many changes are tolerated, and in some cases the effects of changes are suppressed by other changes in the *pac *sequences. In some cases it is possible to rationalize the suppression in terms of the structure shown by mfold [15]; in other cases it is not clear why the suppression is effective. In Φ6, we found little suppression due to changes in the *pac *sequences but we found suppressors by mutation in the sequence of P1, the major structural protein of the core. Genomic RNA that is bound to procapsids can be cross-linked to protein P1 [16]. So far, we have not found suppressors of this type in Φ8 for changes in the *pac *sequences. However, we did obtain a suppressor mutation for another type of packaging program transgression. We found that we could exchange the *pac *sequence of L with that of M. The L segment could be packaged in this case if an amino acid change was present in P1.

The experiments involving manipulation of the *pac *sequence of segment S indicate that the Φ8 packaging mechanism has a lower level of stringency than that of Φ6 [13]. Is there a benefit of low stringency in packaging for a viral system? It appears that the answer is yes in many cases. Many bacteriophages with DNA genomes are able to package host or plasmid DNA and consequently carry out transduction of host genes [17]. Low stringency offers the possiblility of the acquisition of host RNA transcripts by RNA viruses. We have seen that Φ6 can acquire plasmid transcripts that do not have *pac *sequences, albeit at extremely low frequencies [18]. It also allows the acquisition of genomic segments of distantly related phages. Although Φ6 cannot acquire the genomic segments of Φ13 a related cystovirus, Φ13 can acquire the S and M segments of Φ6 [19]. Influenza virus is now believed to have specific packaging of its genomic segments [20]. However the packaging is of low enough stringency that virions can acquire genomic segments from viruses that infect very different host organisms [21].

## Conclusion

The sequences that are necessary for packaging transcripts of the dsRNA genome of bacteriophage Φ8 are located in the 5' untranslated regions of the three genomic segments. Genomic packaging in Φ8 is much less stringent than that found for the distantly related bacteriophage Φ6. Many changes in the *pac *region of S are tolerated while others are suppressed by compensating changes in the RNA sequence. Changes in the packaging program can be produced by mutations that change the amino acid sequence of P1, the major structural protein of the procapsid.

## Methods

### Bacterial strains and plasmids

*Pseudomonas syringae *HB10Y derivative LM2489 was the host for Φ8 [1,19]. *Escherichia coli *strain JM109 (*recA1, endA1, gyrA96, thi, hsdR17, supE44, relA1, λ-, Δ(lac-proAB)*), [F', *traD36, proAB, lacIq, ΔM15*] [22] was used for the propagation of all plasmids. Epicurian Super Competent cells of *E. coli *(Stratagene) were used for transformation after directed mutagenesis. Plasmids utilized in this study are listed in tables 1, 2 and 3. All cDNA plasmids used in this study have a SP6 promoter upstream of the cDNA sequences. The SP6 promoter is preceded by a *BglII *site. The sequences of the Φ8 genomic segments are in GenBank with accession numbers NC_003299, NC_003300 and NC_003299 for L, M and S respectively.

### Reverse genetics for the modification of the viral genome

The simplest means of virus construction is to electroporate host cells with SP6 promoter plasmids that contain cDNA copies of the genomic segments along with a plasmid that codes for SP6 RNA polymerase. We generally add about 200 ng of each cDNA plasmid to about 2 × 10^9 ^cells. Using *colE1 *based plasmids such as pT7T319U, that transcribe but do not replicate in pseudomonads we can produce hundreds of plaques. If the polymerase plasmid is resident in the host strain, we can produce tens of thousands of infectious centers with wild type constructions. Plasmid pLM2790 is a derivative of shuttle vector pLM254 with SP6 polymerase gene of PSR3 [23] cloned into the *BamH1 *site [24]. In cases where we alter the *pac *sequences, the number of plaques can be reduced drastically. In some cases, the resulting phage contain suppressor mutations or reversions of the directed mutations. Although the 5' sequence of the Φ8 plus strands, GAAA, is more compatible with the specificity of SP6 polymerase, it is possible to use T7 RNA polymerase as well, although with somewhat lower efficacy.

If one of the genomic segments has a gene for kanamycin resistance, *kan*, inserted into either its non coding region or replacing one or more genes, then electroporation can result in the establishment of a carrier state in which the viral genome is replicated in cells without lysis and with the expression of the drug resistance so as to form stable drug resistant colonies [14].

### Production of deletions in the *pac *sequences of the Φ8 segments

In some cases, deletions could be made by simply ligating the products of restriction enzyme cuts at unique sites. In other cases, we used oligos that contained a *Pmel *site that occurs uniquely in S at 160 and a jump to a vector sequence to PCR on cDNA copies of segment S. These pieces were ligated to *Pmel *and *EcoRI *in the vector to form the deletions. To make smaller deletions or base changes we used the Quick Change protocol of Stratagene (La Jolla, CA) with oligos listed in Table 5 along with their complements, which involves PCR on a plasmid template and subsequent treatment with *DpnI *to eliminate the template DNA. Changes in segment L usually involved PCR with linkage to the *NsiI *site at position 328. Changes in segment M involved deletions at restriction sites.

### The placing of the *pac *sequence of L on segment M

The *BglII*/*XhoI *fragment of plasmid pLM2669 contains the *pac *sequence of Φ8M as well as the SP6 promoter. This fragment was exchanged with the *BglII*/SmaI fragment of plasmid pLM2653 which carries the cDNA copy of Φ8L (Fig. 1).

### RTPCR

dsRNA was isolated from crude cell lysates by first treating with DNase, then phenol extraction and electroelution of the RNA from agarose gels. The RNA was then denatured and annealed to primer and incubated with AMV reverse transcriptase. The products were then subjected to PCR with Pfu turbo DNA polymerase (Stratagene) [19]. The resulting DNA products were cloned into pT7T319U and sequenced at the Molecular Resources Facility of the University of Medicine and Dentistry of New Jersey.

### Mfolds of segment S *pac *sequences

The secondary structures of the *pac *sequences were determined on the mfold web server using the mfold 3.1 program of Zuker and Turner [15]. The folds were constrained so that the maximum distance between paired bases was 50. The mfold structural predictions for the Φ6 *pac *sequences were previously found to be similar to those found by nuclease sensitivity [10]. The structures shown in Figures 2 and 3 are not unique in that there are several structures with similar free energy values. However, stem-loop F in Figure 2 is present in all of them.

## Authors' contributions

Both JQ and XQ devised, carried out and analyzed the directed changes in the sequences of the viral RNA molecules. LM conceived the project and drafted the manuscript. All authors read and approved the final manuscript.

## Figures and Tables

**Table 1:** Plasmids used in this study with cDNA copies of Φ8L

**Plasmid**	**Changes**	**Oligo**	**Plaques**
2653	wt		>1000
3316	Δ244–328	897	>1000
3172	Δ219–328	801	40
3322	Δ210–328	898	4
3318	Δ185–328	899	0
3320	Δ170–328	900	0
2835/6	Δ135–333		0

**Table 2:** Plasmids used in this study with cDNA copies of Φ8M

**Plasmid**	**Change**	**Oligo**	**Plaques**
2669	wt		>1000
3324	Δ216–219		0
2833	Δ196–215		2
3348	CCA240GC	925	>1000
3356	Δ238–244	925	0
3043	Δ34–39	750	10 turbid

**Table 3:** Plasmids used in this study with cDNA copies of Φ8S

**Plasmid**	**changes**	**oligo**	**plaques**	**suppressors**
2755	wt		600	
2816	Δ137–158	590	>1000	
2817	Δ120–158	591	1	
2819	Δ101–158	592	1	
2820	Δ75–158	593	2	
2914	UAG135CUU	681	400	no
2916	AG83UU	683	1000 small	no
2918	UUCG41Δ	685	200	no
2920	UUCG41CCCA	687	300	no
2929	UU128CA	693	>1000	
2938	A166C	699	251 small	no +A172C* or U161A
2939	UAAA163CCCC	701	207	no + C167U
2955	GG159AU	708	60	no + U135C
				U133A
				G175A
2956	GG159AU	708	>1000	no +A166Δ
	A170 Δ			
2979	GG159AU	708	24	no + C132A
	A166C	714		
2980	GG159AU	708	6	no + C132U
	A166C	714		
2990	GG159AU	708	>1000	
	A166C	714		
	C132U	722		
2997	GG159AU	708	10 small	
	Δ137–158	728		
	A166C	714		
2996	GG159AU	724	700 small	
	Δ137–158	728		
3000	C132U	722	>1000	
2999	C132U	722	0	
	Δ135–143			
3013	GAG138CUC	742	3	no or G111A or U114C
3015	GAG138Δ	744	>1000	
3016				
2940	C199A	703	>1000	

* suppressor containing plaques were larger than the others

**Table 4:** Production of phage by electroporation of cDNA plasmids with *pac *sequence of L in segment M

**Comment**	**M+M*pac***	**M+L*pac***	**L wt**	**L mut**	**S**	**Plaques**
Normal	+		+		+	>1000
M has L*pac*		+	+		+	1 or 0
Mutant L and M having L*pac*		+		+	+	>1000
Mutant L and Normal M	+			+	+	>1000

**Table 5:** oligonucleotides used for mutagenesis of *pac *sequences

**Oligonucleotide**	**Sequence**
590	TCTCAGTTTAAACCTAGAGACAAAGCTGAC
591	TCTCAGTTTAAACCGAGATCCCAGGAAATG
592	TCTCAGTTTAAACCACGAATCACCCCGAGC
593	TCTCAGTTTAAACCTTCGCGCCTTTCAGGG
681	GAGTCAGCTTTGTCTCCTTGAGACTGAGCGTTCGG
683	GGCGCGAAGGTCCCCACCTTCTCGGGGTGATTCGT
685	TTCGCTGGCATAGCTCGAGTGAAGCCTTCCCTG
687	CGCTGGCATAGCTCCCCAGAGTGAAGCCTTCCCTG
693	GATCTCGGAGTCAGCTCAGTCTCTAGGAGACTG
699	GTTCGGTCTCAGGTTTAACCTGAGATTGAG
701	GTTCGGTCTCAGGTTCCCCCTGAGATTGAG
703	GACAATGGGTAGAATATTTCAACTGTTGATGC
708	GTTCGGTCTCAATTTTAAACTGAGATTGAG
714	GTTCGGTCTCAATTTTAACCTGAGATTGAG
722	GTCAGCTTTGTTTCTAGGAGACTGAGCGTTCGG
724	CTTTGTCTCTAATTTTAAACTGAGATTGAG
742	CAGCTTTGTCTCTAGCTCACTGAGCGTTCGGTC
744	CAGCTTTGTCTCTAGACTGAGCGTTCGGTC
750	CGGCAATAAGGGTGGCAAAGAGGGTCGAGC
801	CCCATGCATGATCGCGATGAACGCGAAATGAAAC
897	CCCATGCATAACTCCTATTATTAACTATTAATTG
898	CCCATGCATGAACGCGAAATGAAACTTATCTAATCAC
899	CCCATGCATCACTTGTTATGTGATCAGCTTTTGAGTG
900	CCCATGCATCAGCTTTTGAGTGAAGTGGAAACGGG
925	TAGCTACTAGACAGAAAGCTTCCTAACAAGGAGATGCAC
